# Early Prediction of COVID-19 Associated Hospitalization at the Time of CDC Contact Tracing using Machine Learning: Towards Pandemic Preparedness

**DOI:** 10.21203/rs.3.rs-3213502/v1

**Published:** 2023-08-07

**Authors:** Chen Liang, Tianchu Lyu, Sharon Weissman, Nick Daering, Bankole Olatosi, Neset Hikmet, Xiaoming Li

**Affiliations:** University of South Carolina; University of South Carolina; University of South Carolina; Prisma Health; University of South Carolina; University of South Carolina; University of South Carolina

**Keywords:** COVID-19, contact tracing, machine learning, medical claims, medical record linkage

## Abstract

**Objective:**

To develop and validate machine learning models for predicting COVID-19 related hospitalization as early as CDC contact tracing using integrated CDC contact tracing and South Carolina medical claims data.

**Methods:**

Using the dataset (n=82,073, 1/1/2018 - 3/1/2020), we identified 3,305 patients with COVID-19 and were captured by contact tracing. We developed and validated machine learning models (i.e., support vector machine, random forest, XGboost), followed by multi-level validations and pilot statewide implementation.

**Results:**

Using 10-cross validation, random forest outperformed other models (F1=0.872 for general hospitalization and 0.763 for COVID-19 related hospitalization), followed by XGBoost (F1=0.845 and 0.682) and support vector machine (F1=0.845 and 0.644). We identified new self-reported symptoms from contact tracing (e.g., fatigue, congestion, headache, loss of taste) that are highly predictive of hospitalization.

**Conclusions:**

Our study demonstrated the feasibility of identifying individuals at risk of hospitalization at the time of contact tracing for early intervention and prevention.

**Policy implications:**

Our findings demonstrate existing promise for leveraging CDC contact tracing for establishing a cost-effective statewide surveillance and generalizability for nationwide adoption for enhancing pandemic preparedness in the US.

## 1. Introduction

As of May 30, 2023, COVID-19 has claimed 1.1 million deaths in the United States (US), according to the COVID-19 Data Tracker administrated by the US Centers for Disease Control and Prevention (CDC)^1^. According to the World Health Organization (WHO) COVID Dashboard globally, the number of deaths is 6.9 million^2^. Among the severity levels of SARS-CoV-2 infection ^3^, COVID-19 associated hospitalization exhibited the highest mortality rate, ranging from 20.7% (March 2020) to 2.7% (April 2022), according to the analysis by the National Center for Health Statistics (NCHS).^1^ Existing therapeutics is most effective for individuals at early stage of disease. Data from clinical trials suggested that a number of antiviral agents show the most therapeutic benefits for patients who have not required hospitalization and Intensive Care Unit (ICU); and that combined antiviral agents (e.g., monoclonal antibody) must be administrated at the earliest stage of infection to show therapeutic benefits ^4^.

These findings confirmed a crucial role of early detection of at-risk individuals in order to provide timely medical treatment and proactive intervention. Nevertheless, early detection of individuals who are at risk of disease progression and hospital admission remains an understudied and unaddressed research question. A handful of studies have demonstrated the feasibility of predicting COVID-19 progression ^5,6^, severity levels ^7^, and mortality ^8^ using in-hospital records in conjunction with historical medical records.

Despite robust methodology, such approaches may be limited in terms of clinical usability and plausibility if the prediction cannot identify at-risk individuals early before irreversible adverse disease progression ^4^. A recent systematic review of 731 studies focusing on predictive models for COVID-19 diagnosis and clinical progression and outcomes suggested overly optimistic performance but limited value for clinical practices. One of the root causes is the fact that the selection of predictive model input and output was often determined by data that are available rather than data that are clinically meaningful for medical specialists and public health practitioners ^9^.

A predictive model to identify individuals at risk of hospitalization is highly valuable for effective early therapeutics and intervention, as well as optimization of healthcare resources. These predictive models are potentially valuable for the public health preparedness of future pandemics ^10^. With respect to predictive modeling, the earlier the risk factors can be identified for predicting hospitalization the more valuable such a model would be for early intervention. However, this approach has been limited by the lack of data prior to a recorded hospital admission (i.e., time of output in a predictive model). An important source of data is contact tracing records administrated by CDC. In the United States and many other countries, contact tracing has been a key public health surveillance and base of evidence for timely public health intervention and strategies ^11,12^. However, data collected from contact tracing have not been successfully used for making prediction for at-risk individuals, in part due to limited variables from contact tracing and the nature of contact tracing data being self-reported. Studies using healthcare utilization data alone, such as electronic health records (EHR), can be used to make prediction of severe COVID-19 outcomes at point of hospital admission ^13^, but such predictive models are limited in interpreting risk factors outside of EHR (e.g., self-reported symptoms, occupation, geographic patterns, social determinant of health). We argue that a better practice would be an integration of medical records together with contact tracing, which could harness the power of contact tracing by detecting risk factors for severe disease outcomes as early as at the time of exposure an individual to COVID-19 (i.e., admission of contact tracing). However, such data integration has been challenging due to gaps with respect to the Health Insurance Portability and Accountability Act (HIPAA) restrictions, state data privacy regulations, privacy-preserving record linkage (PPRL) techniques ^14^, uneven testing supply by geographic areas, and inconsistent contact tracing coordination in local states ^15^.

In this study, we reported design, development, multi-level evaluation of machine learning (ML) predictive models that can identify individuals who would progress to COVID-19 associated hospitalization at the time of contact tracing administration or earlier. We used a National Institute of Allergy and Infectious Diseases (NIAID) funded state-wide surveillance dataset that incorporates medical claims and contact tracing administrated through South Carolina (SC) Department of Health and Environmental Control (DHEC). The overarching goal of this study responds to the NIAID’s plan for pandemic preparedness, aiming for expanding the pre-clinical infrastructure capacity ^10^.

[1] An in-hospital death is defined by an encounter with a discharge status of died or died in a medical facility. Data are UB-04 administrative claims data, not nationally representative.

## 2 Methods

### 2.1 Data source

Funded by NIAID, we used a multi-center dataset that integrates SC outpatient and inpatient medical claims data and CDC contact tracing data [i.e., Person Under Investigation (PUI)], with which the data are linked at the individual level by using unified patient IDs determined by SC Revenue and Fiscal Affairs (RFA). Using this dataset, we were able to identify individuals who have COVID-19 related hospitalization and were previously captured by contact tracing between March 01, 2020, and December 31, 2020, and retrieved their sociodemographic data and medical history from two years before the time of the identified hospitalization (i.e., predictive model output).

To establish a cohort of interest, we used the following inclusion criteria 1) individuals who had hospitalization records from medical claims, 2) individuals who were previously captured by contract tracing. The only exclusion criterion was a hospitalization dated before contact tracing records. All medical claims data from two years before a hospitalization record were retrieved.

### 2.2 Machine learning model design

#### Model outputs.

Based on the linked contact tracing and medical claims data, we split the cohort into two datasets, which then became two different ML models with different outputs. For one dataset, the output of ML model was determined by whether a patient was hospitalized after contact tracing, namely, “general hospitalization set”. For another dataset, the output of the ML model was determined by whether a patient was hospitalized caused by COVID-19, namely, “COVID-19 related hospitalization set”. COVID-19 related hospitalization is defined as the hospitalization of patients with a documented positive COVID-19 record using ICD-10 code U07.1.

#### Model inputs (feature space).

After data cleaning, there were 941 features. Sex was represented by female or male. There were seven classes for the race/ethnicity including non-Hispanic White, non-Hispanic Black, Hispanic/Latino, non-Hispanic Asian, non-Hispanic American Indian, other race, and missing value, corresponding to data collected from contact tracing. Age was divided into six age groups:18–29, 30–39, 40–49, 50–59, 60–69, and 70 or older. From medical claims data, the insurance payor variable was categorized into nine groups: out-of-pocket (self-pay), Medicare, Medicaid, commercial insurance, workers compensation, indigent/charitable organization, other government, Health Maintenance Organization (HMO), and not stated. The location variables included 46 counties in SC, representing the residential location of a patient. The symptom variables extracted from the PUI form included nasal congestion, fatigue, fever, chills, myalgia, running nose, sore throat, cough, breathing difficulty, etc., and medical conditions extracted from medical claims data including primary hypertension, type 2 diabetes mellitus without complications, morbid (severe) obesity due to excess calories, short of breath, etc. It is worth noting that some symptoms from contact tracing overlap with those from medical claims data. For example, cough, fever, and pneumonia appeared in both contact tracing and medical claims data, but were treated as different features in the models because they were extracted from different sources and using different data collection methods. For example, symptoms from contact tracing are self-reported whereas symptoms from medical claims are documented by providers. There were 25 symptom variables extracted from contact tracing and 847 medical conditions from medical claims data.

#### Algorithms

We applied supervised ML models to determine predictors of hospitalizations using algorithms including Random Forest (RF) (max depth = 10), Support Vector Machine (SVM) (kernel = radial basis function, gamma = 0.01), and XGBoost (XGB) (max depth = 10). Selection of algorithms represents a collection of well performed algorithms that have been tested on patient data (e.g., medical claims, health administrative data, and electronic health records). To retain the generalizability of tested machine learning models, we only did not alter algorithms other than their parameters.

### 2.3 Evaluation

We developed a stream of approaches to validate the models including mathematical and clinical validation. The mathematical validation consisted of the following steps: First, we used 10-fold cross-validation as validation framework. Using this method, we randomly created 10 splits of data. In each of the 10 iterations, nine splits of data were used for model training and the remaining one split was used for testing, without overlapping with any other iterations. Second, the performance of the models was evaluated using the area under the receiver operating characteristic curve (AUROC), precision, recall, and F1 score. Third, we examined the feature importance using SHapley Additive explanations (SHAP).

The clinical validation included two major steps. Step one was designed to validate the consistency between machine and clinical experts on individual ML features. Based on the SHAP results, two clinical experts (SW and ND) independently rated the top 20 significant features identified by SHAP using a Likert scale of [−5, 5] for each set of models. Being rated as −5 meant that the feature was very likely to be contributing to non-hospitalization; and being rated as 5 meant that the feature was very likely to be contributing to hospitalization. The robustness of the two ranked lists from the experts were assessed by the Content Validity Index (CVI). Then, we calculated the mean values between the ratings of the features from the two experts and scale the corresponding SHAP values to [−5, 5], and compared the mean rating to the scaled SHAP values by Spearman’s rank correlation coefficient and Mann-Whitney U test. Step two was designed to test to what extent clinical experts’ prediction is consistent with machine’s prediction (an individual to be hospitalized or not) upon reviewing a masked real-world patient case from our dataset. We randomly selected five cases from each quadrant of the performance matrix [i.e., false positive (FP) cases, false negative (FN) cases, true positive (TP) cases, and true negative (TN) cases] for each set of models. The narrative summary of a case consisted of machine ranked top-20 features, then polished by ChatGPT for better linguistic nuances. These cases were assigned to the two clinical experts, and each case was rated independently based on a Likert scale of [0, 5], with 0 representing least likely and 5 representing most likely that the patient would be hospitalized. The interrater reliability was evaluated by CVI. Then we calculated the mean of rating for each quadrant between the two experts. We considered the quadrant to be negatively correlating to machine prediction if the mean value was smaller than or equal to 2.5 and to be positively correlating to machine prediction if the mean value was greater than 2.5. Supplement 1 demonstrates the Likert scale design for both steps of clinical evaluation as well as narrative summaries of cases.

## 3 Results

In a total of 82,073 individuals in our dataset, we identified 3,305 patients who had COVID-19 and were previously captured by contact tracing. Among these patients, 2,022 individuals were at least admitted into the inpatient setting once and 217 patients had at least one COVID-19 related hospitalization. Among all the patients, female patients accounted for 60% and male patients accounted for 40%, with similar distribution when being stratified by COVID-19 infection. White patients were the largest racial group in the cohort (54.6%), followed by Black patients (37.1%), Hispanic/Latino patients (4.9%), and other races. In COVID-19 positive group, Black patients accounted for 52.4%, followed by White patients (30.0%), Hispanic/Latino patients (11.9%), and other races. Table 1 is the sociodemographic description of the data set.

**Figure 1.**
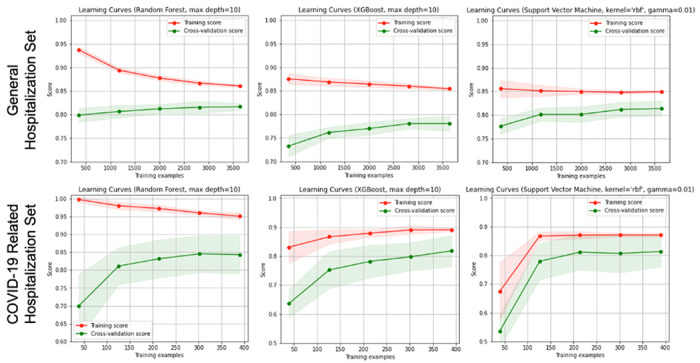
Learning curves of the models.

Two sets of ML models were fitted for making predictions, where each set was trained using three algorithms (i.e., RF, XGB, SVM). The first set (i.e., general hospitalization set) of models specified whether the patient had been admitted to the hospital as the output (n = 2,022 for positive), while the second set (COVID-19 related hospitalization set) of models specified whether the patient had COVID-19 related hospitalization as the output (n = 217 for positive). These 2,022 and 217 individuals, respectively, were matched with control cases at a ratio of 1:1 to balance the data in each set of models. With 10-cross validation, RF outperformed other models (F1 = 0.872 for the general hospitalization set and 0.763 for the COVID-19 related hospitalization set), followed by XGB (F1 = 0.845 and 0.682, correspondingly) and SVM (F1 = 0.845 and 0.644, correspondingly). See Table 1 for numeric results. As displayed in Fig. 1, the learning curves demonstrated the impact of underfitting is minimized for finalized model to be trained.

**Figure 2.**
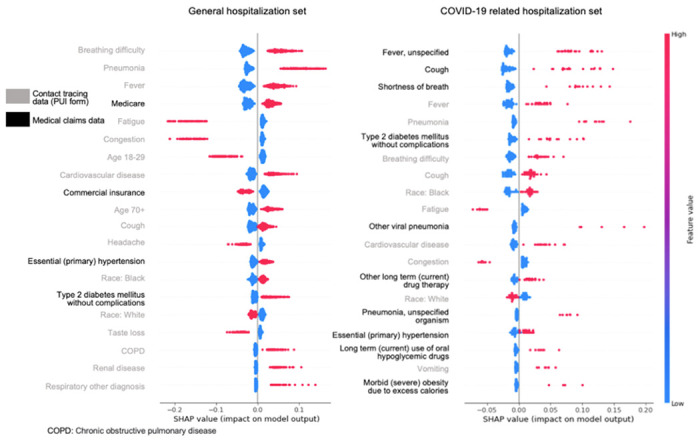
Feature ranking and importance magnitude using SHAP for general hospitalization set (left) and COVID-19 related hospitalization set (right). Grey legends in y-axis represent contact tracing data; blacked legends represent medical claims data.

Among patients admitted to inpatient settings during the COVID-19 pandemic (general hospitalization set), our findings revealed that the most significant predictors of hospitalization are from the contact tracing data, which included breathing difficulty, fever, pneumonia, cardiovascular disease, cough, chronic obstructive pulmonary disease (COPD), renal disease, and other respiratory conditions (Fig. 2). Notably, features such as fatigue, congestion, headache, and loss of taste from contact tracing were associated with a lower likelihood of hospitalization. Additionally, demographic factors such as being aged 70 or older and Black race were positively correlated with hospitalization, whereas younger age (18–29) and White race showed a negative association. Features from medical claims data including Medicare coverage, primary hypertension, and type 2 diabetes mellitus without complications were associated with a higher risk of hospitalization, while commercial insurance coverage indicated a lower risk of hospitalization. Among all the features, the top three most predictive factors were breathing difficulty, pneumonia, and fever.

For patients who experienced COVID-19 related hospitalization (COVID-19 related hospitalization set), features from medical claims data played a more prominent role than the other set (Fig. 2). The top positive contributors from medical claims data to COVID-19 related hospitalization were fever (unspecified), cough, shortness of breath, type 2 diabetes mellitus without complications, other viral pneumonia, other long-term (current) drug therapy, pneumonia (unspecified organism), primary hypertension, long-term (current) use of oral hypoglycemic drugs, and morbid (severe) obesity due to excess calories. From the contact tracing data, the positive features that significantly contributed to hospitalization were fever, pneumonia, breathing difficulty, cough, Black race, cardiovascular disease, and vomiting. Conversely, the three highest-ranked negative features, all derived from the contact tracing, were White race, fatigue, and congestion, suggesting individuals with these features are less likely to be hospitalized.

Results from step one clinical evaluation reached a CVI of 0.7 for general hospitalization set and 0.9 for COVID-19 related hospitalization set, indicating a fair to good agreement. We set a significance level of 0.05 for the tests. The Spearman’s rank correlation coefficients between the mean rating and the scaled SHAP values for the top 20 features were 0.57 with a p-value of 0.009 and 0.52 with a p-value of 0.019 correspondingly, suggesting a significant positive correlation. The Mann-Whitney U test statistics were 199.0 with a p-value of 0.998 and 191.5 with a p-value of 0.828 correspondingly, indicating there was no significant difference between the two ranked lists.

For the step two clinical evaluation, the mean values for FN, FP, TN, and TP were 1.7, 4.1, 2.1, and 3.8, respectively, for the general hospitalization set, which are in alignment with the ML results. The mean values for FN, FP, TN, and TP were 2.5, 2.6, 2.6, and 3.2, respectively, for the COVID-19 related hospitalization set, which remain in alignment with the ML results, but the effect is weaker than the other data set.

**Figure 3.**
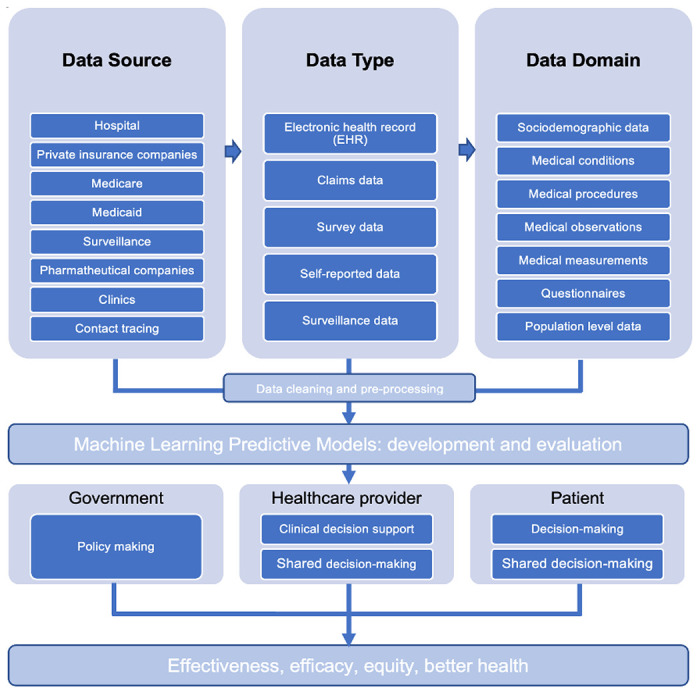
Advanced care planning framework for patients in South Carolina.

Based on the best-performed RF models, we identified the most robust predictors for those with COVID-19 exposure and progressed adversely to hospitalization, which include underlying conditions such as primary hypertension, cardiovascular disease, type 2 diabetes mellitus, pneumonia, and nuanced patterns of predictors from both contact tracing and health care history. Two researchers (CL and TL) reviewed these predictors and organized them into several healthcare systems where public health practitioners or clinicians can respond as early as these predictors are detected in individuals. Figure 3 shows a diagram of these findings which was inspired by the Advanced Care Planning framework^16^. Generally, we found that some of the data captured by contact tracing are highly predictive of COVID-19 related hospitalization such as self-reported fever, pneumonia, breathing difficulty, cough, cardiovascular disease, and vomiting. These data were self-reported in this study but in the real world we have ample opportunities to capture these data. For example, cardiovascular disease could be found in contact tracing data, historical claims data, and EHR data (Fig. 3). In addition, individual-reported fatigue and congestion are strongly associated with disease progression that does not lead to the need of hospitalization. SC DHEC can utilize such information to create a surveillance system for prioritizing actionable responses including individual outreach and referral. Some other symptoms, however, have been challenging for the machine to make accurate predictions, such as cough, breathing difficulty, and cardiovascular conditions, which deserve a close investigation considering individuals’ health history, healthcare utilization after contact tracing. Conditions such as viral pneumonia and pneumonia with unspecified organism, fever, cough, and shortness of breath, when diagnosed at clinical sites rather than self-reported at contact tracing are suggestive of COVID related hospitalization. These nuances can be used by SC DHEC and domestic clinical sites for refining risk factors for better surveillance.

## 4 Discussion

### 4.1 Principal findings

This study is among the first to integrate individual-level contact tracing data with statewide medical claims to identify individuals who will be hospitalized due to COVID-19 at the time of contact tracing. Our study demonstrated the feasibility of leveraging CDC contact tracing for early detection of at-risk individuals with exposure to COVID-19 in South Carolina, and the potential for nation-wide implementation.

In this study, we tuned and implemented three well-performed ML algorithms (i.e., RF, XGB, and SVM) on two sets of SC data: the general hospitalization set, and the COVID-19 hospitalization set. In general, RF models performed the best with an F1 score of 0.872 for the general hospitalization set and 0.763 for the COVID-19 hospitalization set. Although both F measures indicate good predictive performance, the small difference between the two data sets suggest challenges in predicting COVID-19 related hospitalization as comparing to hospitalization following exposure to COVID-19 but may or may not be clinically attributed to SARS-CoV-2 infection. The most significant predictors for individuals progressing to hospitalization were breathing difficulty, pneumonia, fever, cardiovascular disease, higher age (70 or older), cough, primary hypertension, being Black race, type 2 diabetes, COPD, renal disease, and other respiratory conditions. In contrast, symptoms such as fatigue, congestion, headache, and taste loss, and factors such as being younger age (18–29), commercial insurance coverage, and being White race were associated with a lower likelihood of hospitalization. The most significant predictors for COVID-19 related hospitalization included comorbidities and symptoms such as fever, cough, shortness of breath, pneumonia, type 2 diabetes mellitus, breathing difficulty, cardiovascular disease, primary hypertension, vomiting, and obesity. Individuals of Black race had a higher risk of COVID-19 related hospitalization. In contrast, fatigue, congestion, and White race indicated a lower likelihood of COVID-19 related hospitalization.

In both the general hospitalization set and the COVID-19 hospitalization set, certain features consistently emerged as significant indicators of hospitalization. These features include breathing difficulty, pneumonia, fever, cough, primary hypertension, and type 2 diabetes mellitus. Such findings suggest that these factors are consistently associated with a higher risk of severe illness and the need for hospitalization among individuals with COVID-19 infection during the pandemic. Either providers or other clinicians should be alarmed for early intervention (e.g., patient outreach, referral) when one or more of these signals in self-reported contact tracing data, healthcare utilizations, or other possible data sources. For both models, we found several self-reported symptoms such as fatigue and congestion are strongly predictive of non-hospitalization, which have not been previously reported. The findings of this study need confirmation with prospective evaluation. If the model remains robust in a prospective study this has the potential to be used by SC DHEC and healthcare systems for screening individuals into different risk categories as early as contact tracing data are obtained. However, we should be careful when interpreting these findings. Fatigue and congestion are mild conditions and sometimes can progress to severe symptoms, meaning that without further clinical examination they should not be regarded as independent predictive factors for non-hospitalization.

### 4.2 Policy implications

There have been debates for the effectiveness of contact tracing in measuring and containing COVID-19 for reasons such as timeliness of data reporting and unequal deployment due to different healthcare recourse by geographic locations^17^. Our findings demonstrated the unique advantages of contact tracing data for early identification of at-risk individuals, suggesting existing potentials for public health implementation. However, to enable such predictive analysis, contact tracing data needs to be linked with healthcare utilization and medical records (e.g., medical claims, EHR) at individual level to enrich the data fed to predictive models. In this regard, the S3C dataset has successfully demonstrated the benefits of this approach.

Our study demonstrated a new approach to establishing a cost-effective surveillance system in South Carolina. Establishing a surveillance for either clinical or public health setting would be costly given the exceeding large population base with exposure to COVID-19 in the US. Our study demonstrated how machine-learning models can be advantageous and less costly for making predictions. First, because machine learning models are not assumption based, they can identify highly predictive features without assuming candidate features (e.g., coefficients in parametric statistical models). Second, because machine learning models are sustainable for high dimensional data, they are competent for processing exceedingly large number of variables, as is the norm in large datasets especially when combining dataset such as contact tracing and medical records.

The capability of proactively identifying at-risk individuals as early as contact tracing demonstrates the potential for clinical implementation. This unique advantage not only benefits individuals by ensuring timely care but also preserves the limited healthcare resources during the pandemic by optimizing safety measures and interventions towards for those with urgent medical needs. For example, if an individual exhibits high-risk features identified in this study through historical claims data or EHR, proactive measures can be taken by social workers or other healthcare workers to assess the individual’s health status by telehealth measures, which would reduce the demand for healthcare resources and the risk of community spread. By promptly initiating patient outreach with these individuals, proactive interventions and timely therapeutics can be put into action to mitigate the likelihood of hospitalization.

### 4.3 Limitations

There are some limitations to the presented study. First, because of the data quality for contact tracing and medical claims, we removed 60% of missing values, which reduced the sample size and may exclude a few predictive features with too many missing values. Second, COVID-19 related hospitalization is identified by medical codes, which may have missed clinical visits that have not been appropriately coded. Third, we did not test external validity in this study, which would limit the study findings to be generalizable for different healthcare systems and/or datasets. Despite limitations, this study demonstrates how CDC contact tracing when integrated with state-level healthcare utilization data can be used to predict COVID-19 related hospitalizations at the time of contact tracing, which encourages research opportunities for statewide implementation and nationwide generalizability validation.

## Figures and Tables

**Table 1.** Demographics of the South Carolina cohort by sex, race, age, and hospitalization, stratified by SARS-CoV-2 infection.

		Overall	COVID-19 Negative	COVID-19 Positive	P-value
**n**		82073	78768	3305	-
**Sex, n (%)**	**Female**	49232 (60.0)	47301 (60.1)	1931 (58.4)	0.064
	**Male**	32841 (40.0)	31467 (39.9)	1374 (41.6)	-
**Race, n (%)**	**White**	44789 (54.6)	43796 (55.6)	993 (30.0)	<0.001
	**Black**	30447 (37.1)	28715 (36.5)	1732 (52.4)	-
	**Hispanic/Latino**	3996 (4.9)	3604 (4.6)	392 (11.9)	-
	**Asian**	322 (0.4)	308 (0.4)	14 (0.4)	-
	**American Indian**	143 (0.2)	141 (0.2)	2 (0.1)	-
	**Other**	1256 (1.5)	1139 (1.4)	117 (3.5)	-
	**NA**	1120 (1.4)	1065 (1.4)	55 (1.7)	-
**Age; n (%)**	**18-29**	15841 (19.3)	15068 (19.1)	773 (23.4)	<0.001
	**30-39**	12686 (15.5)	12007 (15.2)	679 (20.5)	-
	**40-49**	12735 (15.5)	12105 (15.4)	630 (19.1)	-
	**50-59**	15423 (18.8)	14826 (18.8)	597 (18.1)	-
	**60-69**	12811 (15.6)	12444 (15.8)	367 (11.1)	-
	**70+**	12577 (15.3)	12318 (15.6)	259 (7.8)	-
**Hospitalization, n(%)**	**No**	80051 (97.5)	76963 (97.7)	3088 (93.4)	<0.001
	**Yes**	2022 (2.5)	1805 (2.3)	217 (6.6)	-

**Table 2.** Model performance by AUROC, precision, recall, and F1 score

	General Hospitalization Set			COVID-19 Related Hospitalization Set		
	RF	XGB	SVM	RF	XGB	SVM
**AUROC**	0.808	0.792	0.851	0.819	0.759	0.743
**Precision**	0.837	0.844	0.876	0.840	0.737	0.835
**Recall**	0.911	0.844	0.817	0.717	0.652	0.539
**F1 score**	0.872	0.845	0.845	0.763	0.682	0.644

## Data Availability

Data dictionary and programming codes are available upon request. Evaluation instruments are made available from supplemental materials.
